# Crystal structure of dimethyl 3,4,5,6-tetra­phenyl­cyclo­hexa-3,5-diene-1,2-di­carboxyl­ate

**DOI:** 10.1107/S2056989016009403

**Published:** 2016-06-14

**Authors:** Fred H. Greenberg, Alexander Y. Nazarenko

**Affiliations:** a Chemistry Department, SUNY Buffalo State, 1300 Elmwood Ave, Buffalo, NY 14222, USA

**Keywords:** crystal structure, 1,3-cyclo­hexa­diene, conformation

## Abstract

In the title compound, C_34_H_28_O_4_, the cyclo­hexa­diene ring has a screw-boat conformation with a torsion angle between the double bonds being on average *ca* 15° [15.2 (3) and −15.3 (3) in the two independent mol­ecules]. All four phenyl rings in both mol­ecules are arranged in a propeller-like conformation. The two mol­ecules exhibit S,*R*- and *R*,*S*- chirality, respectively, and are connected *via* C—H⋯O inter­molecular inter­actions. In turn, these weakly bound dimers form the mol­ecular crystal.

## Abstract 2

In the title compound, C_34_H_28_O_4_, the cyclo­hexa­diene ring has a screw-boat conformation. All four phenyl rings in the two independent mol­ecules are arranged in a propeller-like conformation. The two mol­ecules exhibit S,*R*- and *R*,*S*- chirality and are connected *via* C—H⋯O inter­molecular inter­actions.

## Chemical context   

Addition reactions of tetra­phenyl­cyclo­penta­dienone, often abbreviated to ‘tetra­cyclone’, were reviewed by Allen (1945 ▸, 1962 ▸). Tetra­cyclone reacts with unsaturated anhydrides, acids and esters, forming a number of polyfunctional carbonyl-bridge compounds. These species easily loose carbon monoxide to form di­hydro­benzene (cyclo­hexa­diene) derivatives. It was found that the use of maleic and fumaric esters yields various stereoisomers. The photochemical behavior of these compounds was studied (Fuchs & Yankelievich, 1968 ▸), showing a number of products including dimethyl tetra­phenyl­phthalate. The relative simplicity of these reactions and the rich organic chemistry and spectroscopy of appropriate products make them attractive for use in undergraduate organic chemistry teaching laboratories.
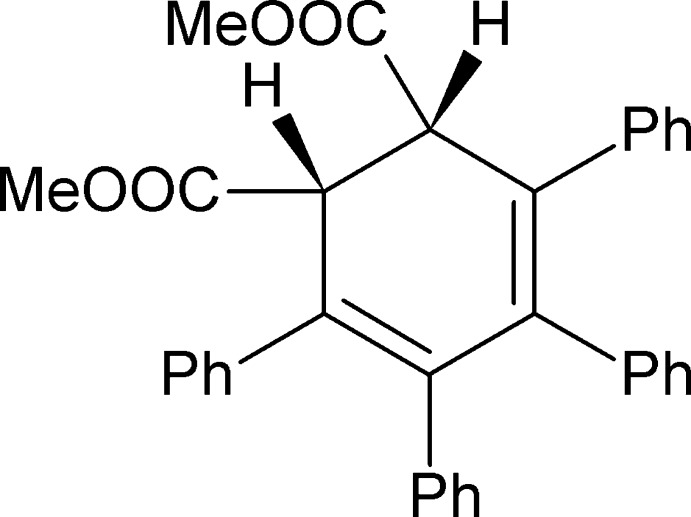


This study provides an opportunity to investigate the geometry of 1,3-cyclo­hexa­diene rings surrounded by bulky substituents with no strong inter­molecular inter­actions.

## Database survey   

Conjugation of two double bonds favors a coplanar π-system with a dihedral angle close to zero. However, in cyclic 1,3-cyclo­hexa­diene mol­ecules angle strain and steric effects promote a non-planar structure (Rabideau & Sygula, 1989 ▸). Even for non-cyclic systems, because of steric effects, the geometry of the higher energy non-trans conformer of 1,3-butadiene in the gas phase is non-planar *s-gauche* (De Maré *et al.*, 1997 ▸). Addition of bulky substituents to the 1,3-butadiene mol­ecule changes the conformational preference from *trans* to *gauche* even in the ground state.

The geometry of unsubstituted 1,3-cyclo­hexa­diene was studied using electron diffraction in the gas phase (Traetteberg, 1968 ▸; Rabideau & Sygula, 1989 ▸) showing a dihedral angle of around 18°. The crystal structure of solid unsubstituted 1,3-cyclo­hexa­diene is not reported. However, the 1,3-cyclo­hexa­diene mol­ecule has been incorporated into microporous vanadium benzene­dicarboxyl­ate (Wang *et al.*, 2011 ▸) showing an almost flat conformation with a dihedral angle of 3.9° (refcode IXODUV). There are a large number of known 1,3-cyclo­hexa­diene complexes with various metals, all with a mostly planar diene fragment. There are seventeen reported hexa­substituted 1,3-cyclo­hexa­diene structures deposited in the Cambridge Structural Database (CSD Version 5.37; Groom *et al.*, 2016 ▸). Of these structures, nine show a practically flat butadiene fragment with dihedral angles less than 3°. Two more (refcodes ONIWUE and TESNIT) show dihedral angles of 4.5 and 4.7°, respectively. Only four structures demonstrate dihedral angles similar to that of free 1,3-cyclo­hexa­diene in the gas phase: GABGEQ (18.8°), HEUZOX (22.5°), JEKFUB (18.6°) and PUBMEG (20.1°). This last structure of *trans*-dimethyl 3,4,5,6-tetra­methyl­cyclo­hexa-3,5-diene-1,2-di­carboxyl­ate (Takahashi *et al.*, 1998 ▸) is the closest to the title compound, with a *cis* conformation as for the title compound.

## Structural commentary   

**Figure 1:**
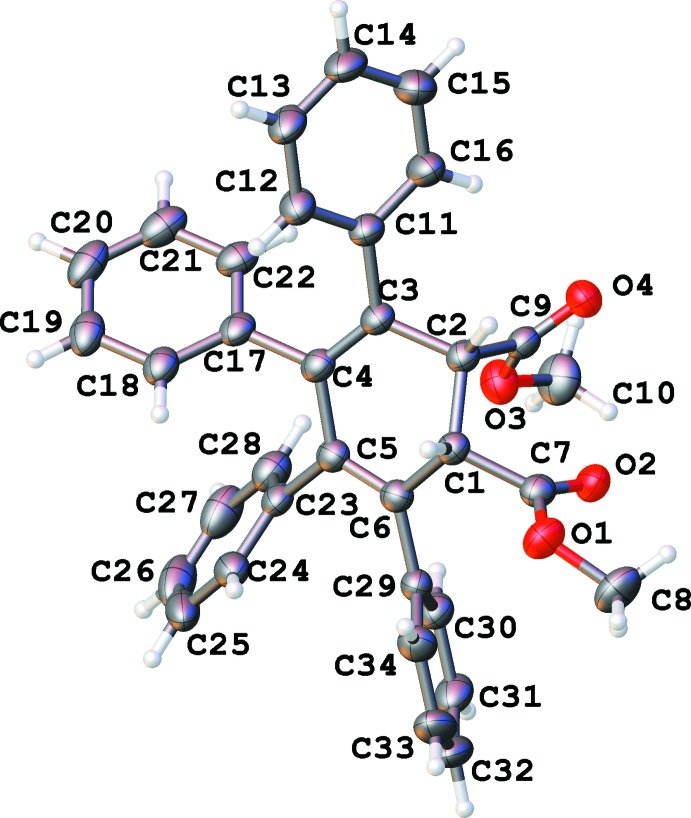
Numbering scheme of the title compound with 50% probability elipsoids (*S*,*R*-isomer).

**Figure 3:**
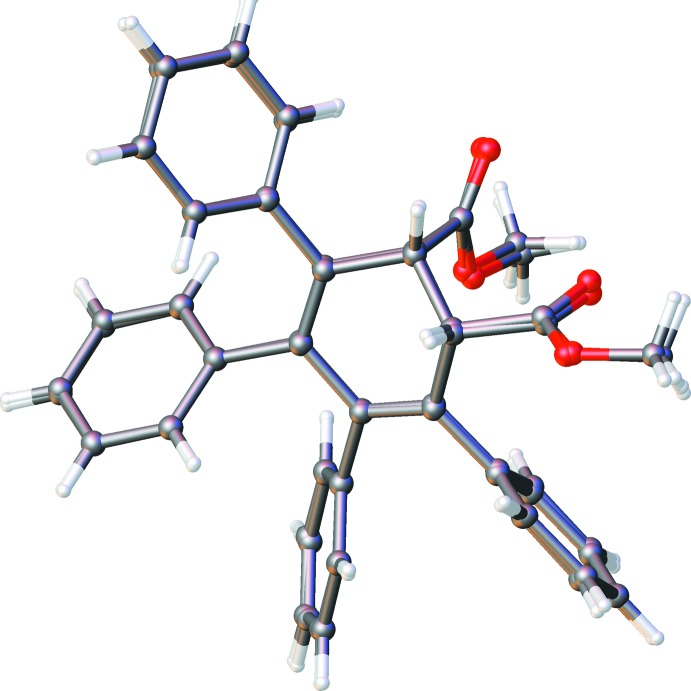
Overlay of the two independent mol­ecules, after inversion.

There are two independent mol­ecules (Figs. 1 ▸ and 2 ▸) in the asymmetric unit of the title compound, with *S*,*R*-chirality and *R*,*S*-chirality, respectively (Figs. 1 ▸, 2 ▸). After inversion they demonstrate a good overlay (Fig. 3 ▸) with an average deviation of 0.14 Å.

**Figure 4:**
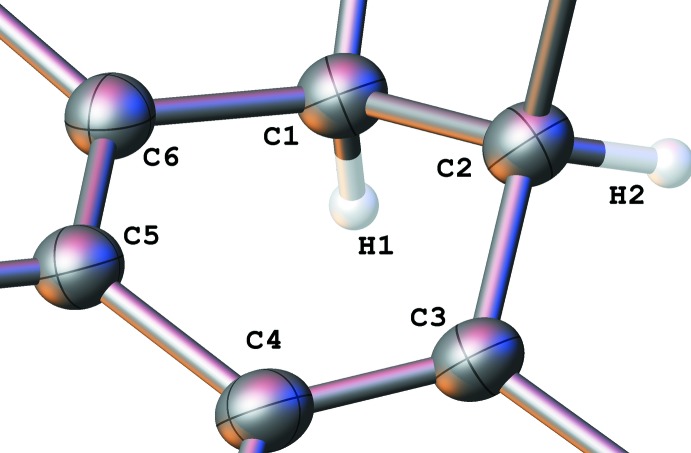
Cyclo­hexa­diene ring with 50% probability elipsoids.

The cyclo­hexa­diene rings (see Fig. 4 ▸, Table 1 ▸) are non-planar in a screw-boat conformation (Boeyens, 1978 ▸) with puckering parameters (C1–C6) *Q* = 0.437 (2) Å, θ = 115.8 (3)° and φ = 213.1 (3); (C101–C106) *Q* = 0.463 (2) Å, θ = 63.7 (2)° and φ = 33.5 (3)°.

Torsion angles between *C*sp^3^ atoms indicate a *gauche* conformation; the dihedral angles between the two double bonds are 15.2 (3) and −15.3 (3) for the two independent mol­ecules (see Table 2 ▸). These values are practically the same as observed for free 1,3-cyclo­hexa­diene in the gas phase: one can argue that the much lower values reported for 1,3-cyclo­hexa­dienes in the crystal state are caused by inter­molecular inter­actions which may favor a flat butadiene fragment.

All six substituents are practically flat. Both ester fragments are almost perpendicular to the mean plane of the cyclo­hexa­diene ring (Table 3 ▸). All four phenyl rings in both mol­ecules are arranged in a propeller-like formation with angles between 46 and 74° (see Table 3 ▸ for exact numbers) from the mean plane of the cyclo­hexa­diene ring. This propeller-like formation is probably inherited from the precursor tetra­cyclone mol­ecule (refcode KIKTUT02; Pal *et al.*, 2014 ▸). Because of the large angles between the planes of the double bonds and each phenyl ring, very little conjugation may be expected. Therefore, substituents serve mainly as bulky decoration, protecting the cyclo­hexa­diene ring from external steric influences.

## Supra­molecular features   

There are no usual hydrogen-bonding or stacking inter­actions in this structure.

**Figure 5:**
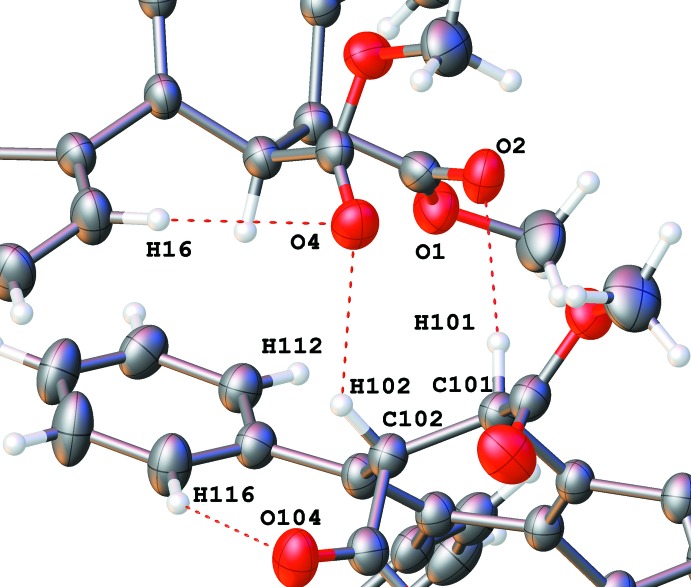
Short C—H⋯O contacts connecting two mol­ecules into a weakly bonded dimer in the crystal.

Two hydrogen atoms of the cyclo­hexa­diene group (H101 and H102) form short contacts (Desiraju & Steiner, 1999 ▸) with carbonyl oxygen atoms of another mol­ecule (Table 4 ▸, Fig. 5 ▸). The corresponding hydrogen atoms of the other mol­ecule (H1 and H2) do not have acceptors available for such bonding. These inter­molecular inter­actions, however weak they are, keep together a pair of mol­ecules with opposite chirality. Two short intra­molecular C—H⋯O contacts within each mol­ecule are also observed and may influence the mol­ecular conformation. There are no other bonding short contacts between the weakly bound dimers and they form a usual mol­ecular crystal.

## Synthesis and crystallization   

The title compound was obtained by reaction of tetra­phenyl­cyclo­penta­dienone (common name tetra­cyclone) with di­methyl­maleate following Allen & Sheps (1934 ▸). GC–MS analysis of the colorless crystalline product dissolved in di­chloro­methane shows one main compound with a parent peak at 500 which is consistent with the formula weight of the title compound. Because all precursor compounds were non-chiral and synthetic conditions should not induce chirality, we expected to see a racemic product. Crystallization from aceto­nitrile resulted in several hexa­gonal flakes, mostly with inter­grown smaller crystals. Several crystals were tested, all resulting in essentially the same chiral trigonal structure. The highest quality structure, from a partial racemically twinned crystal, is reported here.

## Refinement   

Crystal data, data collection and structure refinement details are summarized in Table 5 ▸. The structure was refined as a two-component inversion twin. Cyclo­hexa­diene hydrogen atoms H1, H2, H101 and H102 were refined in isotropic approximation with *U*_iso_ = 1.2*U_i_*_so_(C). All aromatic hydrogen atoms were refined with riding coordinates with C—H = 0.95–0.98 Å and *U*_iso_ = 1.2*U*_iso_(C). Idealized methyl groups were refined as rotating groups with *U*_iso_ = 1.5U_iso_(C).

## Supplementary Material

Crystal structure: contains datablock(s) I. DOI: 10.1107/S2056989016009403/zl2665sup1.cif

Structure factors: contains datablock(s) I. DOI: 10.1107/S2056989016009403/zl2665Isup2.hkl

Supporting information file. DOI: 10.1107/S2056989016009403/zl2665Isup3.cdx

Supporting information file. DOI: 10.1107/S2056989016009403/zl2665Isup4.cml

CCDC reference: 1484412

Additional supporting information: crystallographic information; 3D view; checkCIF report

## Figures and Tables

**Figure 2:**
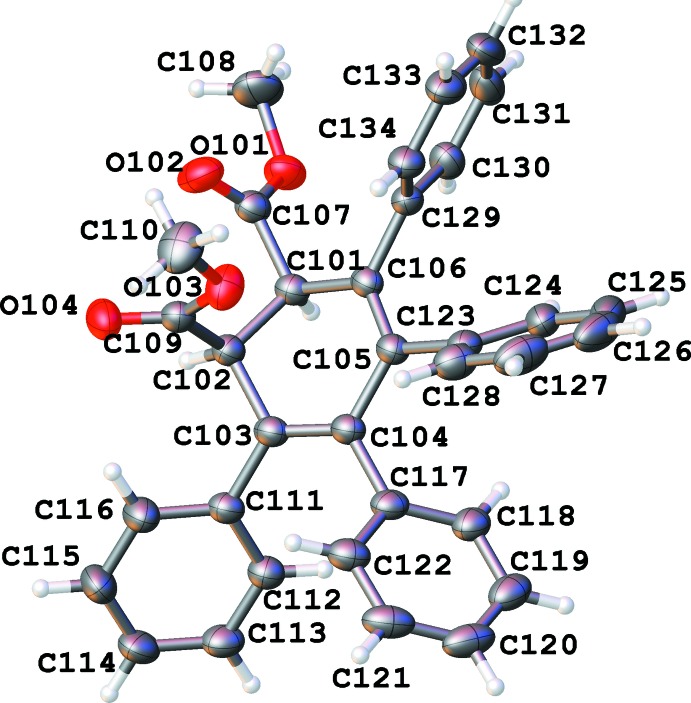
Numbering scheme of the title compound with 50% probability elipsoids (*R*,*S*-isomer).

**Table 1:** Deviation from the mean plane of cyclo­hexa­diene ring (Å)

C1	−0.269 (2)	C101	−0.286 (2)
C2	+0.280 (2)	C102	+0.298 (2)
C3	−0.089 (2)	C103	−0.096 (2)
C4	−0.112 (2)	C104	−0.114 (2)
C5	+0.126 (2)	C105	+0.131 (2)
C6	+0.064 (2)	C106	+0.067 (2)

**Table 2:** Selected torsion angles (°)

C4—C3—C2—C1	−35.7 (3)	C105—C104—C103—C102	−5.2 (3)
C4—C5—C6—C1	0.7 (3)	C5—C4—C3—C2	4.3 (3)
C3—C4—C5—C6	15.2 (3)	C5—C6—C1—C2	−32.9 (3)
C3—C2—C1—C6	48.2 (2)	C106—C101—C102—C103	−51.3 (2)
C101—C102—C103—C104	38.2 (3)	C102—C101—C106—C105	35.2 (3)
C104—C105—C106—C101	−1.3 (3)	C103—C104—C105—C106	−15.3 (3)

**Table 3:** Dihedral angles between cyclo­hexa­diene mean plane and substituent mean planes (°)

Atoms	angle	atoms	angle
C8/O2/C7/O1	79.35 (9)	C108–O101	71.07 (10)
C10/O4/C9/O3	97.38 (13)	C110–O104	97.82 (14)
C11–C16	59.72 (8)	C111–C116	57.22 (8)
C17–C22	46.53 (7)	C117–C122	46.12 (8)
C23–C28	56.38 (8)	C123–C128	56.89 (8)
C29–C34	69.88 (8)	C129–C134	73.46 (8)

**Table 4:** Hydrogen-bond geometry (Å, °)

*D*—H⋯*A*	*D*—H	H⋯*A*	*D*⋯*A*	*D*—H⋯*A*
C101—H101⋯O2	0.99 (3)	2.39 (3)	3.384 (3)	176 (2)
C102—H102⋯O4	0.96 (3)	2.48 (3)	3.242 (3)	136 (2)
C16—H16⋯O4	0.95	2.59	3.407 (3)	145
C116—H116⋯O104	0.95	2.54	3.388 (3)	148

**Table 5:** Experimental details

Crystal data	
Chemical formula	C_34_H_28_O_4_
*M*_r_	500.56
Crystal system, space group	Trigonal, *P*3_2_
Temperature (K)	173
*a*, *c* (Å)	10.8330 (12), 39.169 (5)
*V* (Å^3^)	3980.8 (12)
*Z*	6
Radiation type	Cu *K*α
μ (mm^−1^)	0.65
Crystal size (mm)	0.59 × 0.34 × 0.13

Data collection	
Diffractometer	Bruker Photon-100 CMOS
Absorption correction	Multi-scan (*SADABS*; Bruker,2014 ▸/5)
*T*_min_, *T*_max_	0.669, 0.754
No. of measured, independent and observed [*I* > 2σ(*I*)] reflections	53613, 10773, 10345
*R*_int_	0.043
(sin θ/λ)_max_ (Å^−1^)	0.637

Refinement	
*R*[*F* ^2^ > 2σ(*F* ^2^)], *wR*(*F* ^2^), *S*	0.033, 0.091, 1.05
No. of reflections	10773
No. of parameters	702
No. of restraints	1
H-atom treatment	H atoms treated by a mixture of independent and constrained refinement
Δρ_max_, Δρ_min_ (e Å^−3^)	0.19, −0.15
Absolute structure	Refined as an inversion twin
Absolute structure parameter	0.38 (16)

Computer programs: *APEX2* and *SAINT* (Bruker, 2013 ▸), *XT* (Sheldrick, 2015 ▸), *XL* (Sheldrick, 2008 ▸) and *OLEX2* (Dolomanov *et al.*, 2009 ▸).

## supplementary crystallographic information

### Crystal data

**Table:**

C_34_H_28_O_4_	*D*_x_ = 1.253 Mg m^−^^3^
*M**_r_* = 500.56	Cu *K*α radiation, λ = 1.54178 Å
Trigonal, *P*3_2_	Cell parameters from 9883 reflections
*a* = 10.8330 (12) Å	θ = 3.4–78.4°
*c* = 39.169 (5) Å	µ = 0.65 mm^−^^1^
*V* = 3980.8 (12) Å^3^	*T* = 173 K
*Z* = 6	Plate, colourless
*F*(000) = 1584	0.59 × 0.34 × 0.13 mm

### Data collection

**Table:**

Bruker Photon-100 CMOS diffractometer	10345 reflections with *I* > 2σ(*I*)
Radiation source: sealedtube	*R*_int_ = 0.043
φ and ω scans	θ_max_ = 79.0°, θ_min_ = 3.4°
Absorption correction: multi-scan (SADABS; Bruker,2014/5)	*h* = −13→12
*T*_min_ = 0.669, *T*_max_ = 0.754	*k* = −13→13
53613 measured reflections	*l* = −48→48
10773 independent reflections	

### Refinement

**Table:**

Refinement on *F*^2^	Hydrogen site location: mixed
Least-squares matrix: full	H atoms treated by a mixture of independent and constrained refinement
*R*[*F*^2^ > 2σ(*F*^2^)] = 0.033	*w* = 1/[σ^2^(*F*_o_^2^) + (0.0547*P*)^2^ + 0.4031*P*] where *P* = (*F*_o_^2^ + 2*F*_c_^2^)/3
*wR*(*F*^2^) = 0.091	(Δ/σ)_max_ < 0.001
*S* = 1.05	Δρ_max_ = 0.19 e Å^−^^3^
10773 reflections	Δρ_min_ = −0.15 e Å^−^^3^
702 parameters	Absolute structure: Refined as an inversion twin
1 restraint	Absolute structure parameter: 0.38 (16)

### Special details

**Table:**

Geometry. All esds (except the esd in the dihedral angle between two l.s. planes) are estimated using the full covariance matrix. The cell esds are taken into account individually in the estimation of esds in distances, angles and torsion angles; correlations between esds in cell parameters are only used when they are defined by crystal symmetry. An approximate (isotropic) treatment of cell esds is used for estimating esds involving l.s. planes.
Refinement. Refined as a 2-component inversion twin.1. Twinned data refinement Scales: 0.62 (16) 0.38 (16) 2. Fixed Uiso At 1.2 times of: All C(H) groups At 1.5 times of: All C(H,H,H) groups 3.a Aromatic/amide H refined with riding coordinates: C21(H21), C34(H34), C18(H18), C30(H30), C134(H134), C24(H24), C22(H22), C12(H12), C133(H133), C16(H16), C112(H112), C130(H130), C28(H28), C124(H124), C113(H113), C119(H119), C19(H19), C131(H131), C15(H15), C118(H118), C120(H120), C31(H31), C114(H114), C116(H116), C25(H25), C27(H27), C121(H121), C122(H122), C128(H128), C13(H13), C20(H20), C132(H132), C26(H26), C14(H14), C33(H33), C32(H32), C126(H126), C127(H127), C115(H115), C125(H125) 3.b Idealised Me refined as rotating group: C8(H8A,H8B,H8C), C110(H11A,H11B,H11C), C10(H10A,H10B,H10C), C108(H10D,H10E, H10F)

### Fractional atomic coordinates and isotropic or equivalent isotropic displacement parameters (Å^2^)

**Table:**

	*x*	*y*	*z*	*U*_iso_*/*U*_eq_	
O4	0.79329 (17)	0.30493 (18)	0.54107 (4)	0.0438 (4)	
O101	0.96274 (18)	0.63537 (17)	0.51401 (4)	0.0443 (4)	
O103	1.02631 (18)	0.4316 (2)	0.41723 (4)	0.0475 (4)	
O2	0.56790 (17)	0.38169 (16)	0.51915 (4)	0.0434 (4)	
O1	0.3803 (2)	0.25072 (18)	0.48545 (4)	0.0458 (4)	
O3	0.64592 (18)	0.29005 (17)	0.58294 (4)	0.0452 (4)	
O102	1.09927 (18)	0.5677 (2)	0.48612 (5)	0.0540 (4)	
O104	1.03157 (19)	0.27494 (18)	0.45336 (5)	0.0508 (4)	
C9	0.6769 (2)	0.2477 (2)	0.55396 (5)	0.0345 (4)	
C123	0.7593 (2)	0.5516 (2)	0.37918 (6)	0.0363 (4)	
C29	0.2698 (2)	0.2158 (2)	0.56419 (5)	0.0329 (4)	
C7	0.4665 (2)	0.2687 (2)	0.51152 (5)	0.0355 (4)	
C4	0.4035 (2)	−0.0443 (2)	0.58720 (5)	0.0346 (4)	
C3	0.5035 (2)	−0.0149 (2)	0.56313 (5)	0.0340 (4)	
C23	0.2515 (2)	0.0341 (2)	0.62110 (5)	0.0354 (4)	
C109	0.9775 (2)	0.3424 (2)	0.44350 (6)	0.0356 (4)	
C101	0.8547 (2)	0.4732 (2)	0.46876 (5)	0.0327 (4)	
H101	0.772 (3)	0.452 (3)	0.4837 (7)	0.039*	
C104	0.6822 (2)	0.3191 (2)	0.41216 (5)	0.0333 (4)	
C105	0.7663 (2)	0.4780 (2)	0.41074 (5)	0.0328 (4)	
C107	0.9869 (2)	0.5616 (2)	0.49006 (5)	0.0362 (4)	
C5	0.3298 (2)	0.0406 (2)	0.58914 (5)	0.0333 (4)	
C106	0.8498 (2)	0.5531 (2)	0.43712 (5)	0.0318 (4)	
C102	0.8425 (2)	0.3301 (2)	0.45915 (5)	0.0327 (4)	
H102	0.829 (3)	0.276 (3)	0.4797 (7)	0.039*	
C2	0.5490 (2)	0.1126 (2)	0.53935 (5)	0.0335 (4)	
H2	0.586 (3)	0.095 (3)	0.5182 (7)	0.040*	
C6	0.3366 (2)	0.1245 (2)	0.56296 (5)	0.0322 (4)	
C129	0.9408 (2)	0.7112 (2)	0.43578 (6)	0.0342 (4)	
C11	0.5749 (2)	−0.1004 (2)	0.55719 (6)	0.0371 (4)	
C21	0.4450 (3)	−0.2535 (3)	0.65930 (7)	0.0527 (6)	
H21	0.5199	−0.2513	0.6724	0.063*	
C34	0.3107 (3)	0.3209 (2)	0.58899 (6)	0.0397 (5)	
H34	0.3819	0.3349	0.6052	0.048*	
C18	0.2271 (3)	−0.2583 (2)	0.62137 (6)	0.0418 (5)	
H18	0.1516	−0.2602	0.6086	0.050*	
C103	0.7127 (2)	0.2471 (2)	0.43591 (5)	0.0337 (4)	
C111	0.6241 (2)	0.0917 (2)	0.44298 (6)	0.0359 (4)	
C30	0.1673 (2)	0.1994 (3)	0.54024 (6)	0.0434 (5)	
H30	0.1397	0.1294	0.5228	0.052*	
C134	1.0485 (2)	0.7733 (2)	0.41147 (6)	0.0401 (5)	
H134	1.0651	0.7145	0.3963	0.048*	
C1	0.4199 (2)	0.1303 (2)	0.53089 (5)	0.0326 (4)	
H1	0.357 (3)	0.052 (3)	0.5158 (7)	0.039*	
C17	0.3680 (2)	−0.1561 (2)	0.61388 (5)	0.0375 (4)	
C24	0.1072 (3)	−0.0091 (2)	0.62101 (7)	0.0448 (5)	
H24	0.0548	−0.0382	0.6003	0.054*	
C22	0.4763 (3)	−0.1550 (3)	0.63322 (6)	0.0440 (5)	
H22	0.5728	−0.0861	0.6286	0.053*	
C12	0.4937 (3)	−0.2481 (2)	0.55401 (6)	0.0440 (5)	
H12	0.3930	−0.2941	0.5563	0.053*	
C133	1.1322 (3)	0.9209 (3)	0.40914 (7)	0.0529 (6)	
H133	1.2055	0.9623	0.3925	0.063*	
C117	0.5695 (2)	0.2447 (2)	0.38556 (6)	0.0371 (4)	
C16	0.7221 (3)	−0.0358 (3)	0.55332 (7)	0.0479 (5)	
H16	0.7794	0.0649	0.5551	0.057*	
C112	0.4757 (2)	0.0265 (2)	0.44520 (6)	0.0387 (4)	
H112	0.4305	0.0803	0.4403	0.046*	
C130	0.9202 (3)	0.7994 (2)	0.45808 (6)	0.0429 (5)	
H130	0.8489	0.7587	0.4752	0.051*	
C28	0.3246 (3)	0.0744 (3)	0.65205 (6)	0.0469 (5)	
H28	0.4232	0.1038	0.6526	0.056*	
C124	0.7148 (3)	0.6522 (2)	0.38033 (7)	0.0473 (5)	
H124	0.6850	0.6725	0.4014	0.057*	
C113	0.3938 (3)	−0.1154 (3)	0.45445 (6)	0.0458 (5)	
H113	0.2931	−0.1580	0.4557	0.055*	
C119	0.3685 (3)	0.2182 (3)	0.35267 (7)	0.0509 (6)	
H119	0.2984	0.2446	0.3485	0.061*	
C19	0.1960 (3)	−0.3573 (3)	0.64730 (7)	0.0493 (6)	
H19	0.0997	−0.4267	0.6520	0.059*	
C131	1.0040 (3)	0.9472 (3)	0.45532 (8)	0.0554 (7)	
H131	0.9885	1.0071	0.4703	0.066*	
C15	0.7860 (3)	−0.1173 (3)	0.54693 (8)	0.0566 (7)	
H15	0.8866	−0.0720	0.5445	0.068*	
C118	0.4674 (2)	0.2838 (2)	0.37867 (6)	0.0422 (5)	
H118	0.4654	0.3563	0.3919	0.051*	
C120	0.3719 (3)	0.1143 (3)	0.33279 (7)	0.0559 (7)	
H120	0.3049	0.0701	0.3148	0.067*	
C31	0.1052 (3)	0.2850 (3)	0.54173 (8)	0.0554 (7)	
H31	0.0353	0.2732	0.5254	0.066*	
C114	0.4573 (3)	−0.1955 (3)	0.46184 (8)	0.0539 (6)	
H114	0.4010	−0.2924	0.4685	0.065*	
C116	0.6871 (3)	0.0096 (3)	0.45039 (8)	0.0498 (6)	
H116	0.7878	0.0514	0.4493	0.060*	
C25	0.0393 (3)	−0.0097 (3)	0.65115 (9)	0.0627 (8)	
H25	−0.0591	−0.0381	0.6508	0.075*	
C27	0.2558 (4)	0.0723 (3)	0.68193 (7)	0.0617 (8)	
H27	0.3071	0.1000	0.7028	0.074*	
C121	0.4727 (3)	0.0754 (3)	0.33914 (7)	0.0529 (6)	
H121	0.4751	0.0043	0.3254	0.063*	
C8	0.4058 (4)	0.3808 (3)	0.46891 (7)	0.0588 (7)	
H8A	0.3398	0.3574	0.4497	0.088*	
H8B	0.5041	0.4326	0.4605	0.088*	
H8C	0.3909	0.4403	0.4853	0.088*	
C122	0.5707 (3)	0.1388 (2)	0.36532 (6)	0.0439 (5)	
H122	0.6391	0.1101	0.3696	0.053*	
C128	0.8006 (3)	0.5232 (3)	0.34810 (6)	0.0479 (5)	
H128	0.8302	0.4543	0.3468	0.058*	
C13	0.5581 (3)	−0.3293 (3)	0.54756 (7)	0.0509 (6)	
H13	0.5012	−0.4300	0.5456	0.061*	
C20	0.3049 (4)	−0.3547 (3)	0.66614 (7)	0.0543 (7)	
H20	0.2837	−0.4225	0.6838	0.065*	
C132	1.1094 (3)	1.0066 (3)	0.43078 (8)	0.0598 (8)	
H132	1.1662	1.1073	0.4289	0.072*	
C26	0.1132 (4)	0.0303 (3)	0.68154 (8)	0.0677 (9)	
H26	0.0660	0.0290	0.7021	0.081*	
C14	0.7037 (3)	−0.2644 (3)	0.54406 (8)	0.0543 (6)	
H14	0.7477	−0.3199	0.5397	0.065*	
C33	0.2479 (3)	0.4054 (3)	0.59017 (7)	0.0517 (6)	
H33	0.2762	0.4768	0.6072	0.062*	
C32	0.1450 (3)	0.3866 (3)	0.56682 (8)	0.0562 (7)	
H32	0.1015	0.4440	0.5680	0.067*	
C110	1.1609 (3)	0.4582 (4)	0.40345 (8)	0.0621 (7)	
H11A	1.1830	0.5166	0.3828	0.093*	
H11B	1.1551	0.3673	0.3978	0.093*	
H11C	1.2362	0.5087	0.4204	0.093*	
C10	0.7575 (3)	0.4261 (3)	0.59574 (8)	0.0578 (7)	
H10A	0.7695	0.5023	0.5802	0.087*	
H10B	0.8472	0.4248	0.5972	0.087*	
H10C	0.7314	0.4433	0.6185	0.087*	
C126	0.7558 (3)	0.6936 (4)	0.32010 (9)	0.0718 (10)	
H126	0.7545	0.7419	0.3000	0.086*	
C127	0.7991 (3)	0.5949 (4)	0.31868 (7)	0.0664 (9)	
H127	0.8284	0.5750	0.2975	0.080*	
C115	0.6036 (3)	−0.1331 (3)	0.45941 (9)	0.0614 (7)	
H115	0.6477	−0.1882	0.4640	0.074*	
C108	1.0862 (4)	0.7347 (3)	0.53338 (8)	0.0646 (8)	
H10D	1.1554	0.8085	0.5181	0.097*	
H10E	1.1296	0.6840	0.5441	0.097*	
H10F	1.0572	0.7792	0.5511	0.097*	
C125	0.7142 (3)	0.7225 (3)	0.35077 (10)	0.0658 (9)	
H125	0.6846	0.7915	0.3518	0.079*	

### Atomic displacement parameters (Å^2^)

**Table:**

	*U*^11^	*U*^22^	*U*^33^	*U*^12^	*U*^13^	*U*^23^
O4	0.0354 (8)	0.0405 (8)	0.0537 (9)	0.0178 (7)	0.0057 (7)	0.0050 (7)
O101	0.0495 (9)	0.0365 (8)	0.0416 (8)	0.0175 (7)	−0.0063 (7)	−0.0078 (6)
O103	0.0415 (9)	0.0614 (11)	0.0440 (8)	0.0289 (8)	0.0093 (7)	0.0128 (8)
O2	0.0400 (8)	0.0322 (8)	0.0563 (9)	0.0169 (7)	0.0008 (7)	0.0066 (7)
O1	0.0583 (10)	0.0419 (8)	0.0412 (8)	0.0280 (8)	−0.0065 (7)	0.0010 (7)
O3	0.0464 (9)	0.0379 (8)	0.0429 (8)	0.0148 (7)	0.0022 (7)	−0.0064 (6)
O102	0.0349 (9)	0.0540 (10)	0.0670 (11)	0.0177 (8)	−0.0096 (8)	−0.0159 (9)
O104	0.0415 (9)	0.0425 (9)	0.0731 (11)	0.0245 (8)	0.0025 (8)	0.0080 (8)
C9	0.0395 (11)	0.0308 (10)	0.0378 (10)	0.0210 (9)	0.0015 (8)	0.0038 (8)
C123	0.0285 (9)	0.0275 (9)	0.0447 (11)	0.0079 (8)	−0.0063 (8)	0.0014 (8)
C29	0.0314 (10)	0.0299 (9)	0.0387 (11)	0.0162 (8)	0.0053 (8)	0.0043 (8)
C7	0.0397 (11)	0.0367 (11)	0.0360 (10)	0.0234 (9)	0.0049 (8)	0.0009 (8)
C4	0.0363 (10)	0.0277 (9)	0.0409 (10)	0.0169 (8)	−0.0010 (8)	−0.0003 (8)
C3	0.0368 (10)	0.0275 (9)	0.0407 (10)	0.0183 (8)	0.0017 (8)	0.0001 (8)
C23	0.0407 (11)	0.0270 (9)	0.0424 (10)	0.0199 (8)	0.0063 (9)	0.0054 (8)
C109	0.0333 (10)	0.0300 (10)	0.0405 (10)	0.0136 (8)	−0.0048 (8)	−0.0036 (8)
C101	0.0309 (10)	0.0283 (9)	0.0352 (10)	0.0120 (8)	0.0012 (8)	−0.0001 (7)
C104	0.0298 (9)	0.0275 (9)	0.0386 (10)	0.0113 (8)	−0.0011 (8)	−0.0011 (8)
C105	0.0297 (9)	0.0271 (9)	0.0391 (10)	0.0123 (8)	−0.0010 (8)	0.0006 (8)
C107	0.0366 (11)	0.0293 (10)	0.0372 (10)	0.0122 (8)	−0.0012 (8)	0.0024 (8)
C5	0.0320 (9)	0.0275 (9)	0.0403 (10)	0.0148 (8)	0.0005 (8)	−0.0008 (8)
C106	0.0285 (9)	0.0267 (9)	0.0390 (10)	0.0129 (8)	0.0015 (8)	0.0013 (7)
C102	0.0317 (10)	0.0280 (9)	0.0352 (10)	0.0127 (8)	−0.0014 (8)	0.0018 (8)
C2	0.0366 (10)	0.0293 (10)	0.0371 (10)	0.0185 (9)	0.0029 (8)	0.0003 (8)
C6	0.0297 (9)	0.0285 (9)	0.0379 (10)	0.0141 (8)	−0.0003 (8)	−0.0017 (7)
C129	0.0310 (9)	0.0279 (9)	0.0407 (11)	0.0124 (8)	−0.0076 (8)	−0.0002 (8)
C11	0.0409 (11)	0.0331 (10)	0.0435 (11)	0.0231 (9)	0.0053 (9)	0.0023 (8)
C21	0.0789 (19)	0.0489 (14)	0.0456 (13)	0.0434 (14)	−0.0060 (12)	−0.0020 (11)
C34	0.0435 (11)	0.0334 (10)	0.0439 (11)	0.0206 (9)	0.0057 (9)	0.0020 (8)
C18	0.0512 (13)	0.0328 (10)	0.0444 (11)	0.0232 (10)	0.0078 (10)	0.0023 (9)
C103	0.0296 (9)	0.0273 (9)	0.0407 (10)	0.0117 (8)	−0.0004 (8)	−0.0021 (8)
C111	0.0333 (10)	0.0287 (10)	0.0399 (10)	0.0111 (8)	−0.0013 (8)	−0.0005 (8)
C30	0.0386 (11)	0.0473 (12)	0.0462 (12)	0.0230 (10)	−0.0007 (9)	0.0023 (10)
C134	0.0330 (10)	0.0325 (11)	0.0479 (12)	0.0111 (9)	−0.0018 (9)	0.0046 (9)
C1	0.0341 (10)	0.0282 (9)	0.0362 (10)	0.0162 (8)	0.0013 (8)	0.0001 (8)
C17	0.0475 (12)	0.0311 (10)	0.0413 (11)	0.0253 (9)	0.0055 (9)	0.0008 (8)
C24	0.0429 (12)	0.0338 (11)	0.0601 (14)	0.0211 (10)	0.0096 (10)	0.0063 (10)
C22	0.0540 (13)	0.0405 (12)	0.0479 (12)	0.0314 (11)	−0.0010 (10)	−0.0019 (9)
C12	0.0470 (13)	0.0328 (11)	0.0551 (13)	0.0221 (10)	0.0115 (10)	0.0026 (9)
C133	0.0401 (13)	0.0383 (12)	0.0644 (16)	0.0076 (10)	−0.0075 (11)	0.0143 (11)
C117	0.0306 (10)	0.0291 (9)	0.0405 (10)	0.0065 (8)	−0.0004 (8)	0.0038 (8)
C16	0.0426 (12)	0.0359 (11)	0.0693 (16)	0.0228 (10)	0.0041 (11)	0.0035 (11)
C112	0.0350 (10)	0.0323 (11)	0.0439 (11)	0.0132 (9)	−0.0009 (9)	0.0015 (9)
C130	0.0483 (13)	0.0372 (11)	0.0466 (12)	0.0240 (10)	−0.0086 (10)	−0.0053 (9)
C28	0.0597 (15)	0.0412 (12)	0.0442 (12)	0.0284 (11)	0.0031 (11)	0.0043 (10)
C124	0.0358 (11)	0.0352 (11)	0.0671 (15)	0.0149 (10)	−0.0128 (10)	0.0014 (10)
C113	0.0353 (11)	0.0368 (12)	0.0510 (13)	0.0072 (9)	−0.0015 (10)	0.0045 (10)
C119	0.0336 (11)	0.0499 (14)	0.0525 (13)	0.0083 (10)	−0.0045 (10)	0.0143 (11)
C19	0.0654 (16)	0.0325 (11)	0.0494 (13)	0.0240 (11)	0.0166 (11)	0.0035 (9)
C131	0.0707 (18)	0.0381 (12)	0.0644 (16)	0.0325 (13)	−0.0281 (14)	−0.0136 (11)
C15	0.0479 (14)	0.0536 (15)	0.0787 (18)	0.0331 (13)	0.0109 (13)	0.0082 (13)
C118	0.0325 (10)	0.0364 (11)	0.0482 (12)	0.0100 (9)	−0.0028 (9)	0.0057 (9)
C120	0.0425 (13)	0.0533 (15)	0.0414 (12)	0.0010 (11)	−0.0077 (10)	0.0037 (11)
C31	0.0486 (14)	0.0681 (17)	0.0632 (16)	0.0395 (13)	0.0062 (12)	0.0192 (14)
C114	0.0485 (14)	0.0295 (11)	0.0682 (16)	0.0079 (10)	−0.0093 (12)	0.0084 (10)
C116	0.0372 (12)	0.0345 (12)	0.0738 (17)	0.0150 (10)	−0.0039 (11)	0.0053 (11)
C25	0.0582 (16)	0.0410 (13)	0.093 (2)	0.0279 (12)	0.0374 (16)	0.0202 (14)
C27	0.099 (2)	0.0475 (14)	0.0420 (13)	0.0394 (16)	0.0096 (14)	0.0059 (11)
C121	0.0499 (14)	0.0384 (12)	0.0451 (12)	0.0032 (11)	0.0017 (11)	−0.0038 (10)
C8	0.079 (2)	0.0536 (15)	0.0515 (14)	0.0394 (15)	−0.0100 (13)	0.0079 (11)
C122	0.0402 (12)	0.0330 (11)	0.0464 (12)	0.0093 (9)	−0.0002 (9)	−0.0011 (9)
C128	0.0423 (12)	0.0469 (13)	0.0429 (12)	0.0136 (11)	−0.0050 (9)	−0.0004 (10)
C13	0.0634 (16)	0.0358 (12)	0.0622 (15)	0.0314 (12)	0.0172 (12)	0.0056 (11)
C20	0.090 (2)	0.0389 (12)	0.0417 (12)	0.0383 (14)	0.0087 (12)	0.0054 (10)
C132	0.0591 (16)	0.0268 (11)	0.0795 (19)	0.0111 (11)	−0.0274 (14)	0.0048 (12)
C26	0.102 (3)	0.0474 (15)	0.0592 (17)	0.0412 (16)	0.0416 (18)	0.0158 (13)
C14	0.0666 (17)	0.0505 (14)	0.0664 (16)	0.0446 (14)	0.0177 (13)	0.0105 (12)
C33	0.0654 (16)	0.0380 (12)	0.0602 (15)	0.0322 (12)	0.0197 (13)	0.0059 (11)
C32	0.0607 (16)	0.0552 (15)	0.0737 (17)	0.0447 (14)	0.0236 (14)	0.0229 (13)
C110	0.0464 (14)	0.082 (2)	0.0612 (16)	0.0350 (14)	0.0173 (12)	0.0150 (14)
C10	0.0629 (17)	0.0386 (12)	0.0589 (15)	0.0156 (12)	−0.0056 (12)	−0.0118 (11)
C126	0.0500 (16)	0.0654 (19)	0.0699 (19)	0.0064 (14)	−0.0224 (14)	0.0262 (15)
C127	0.0517 (15)	0.074 (2)	0.0434 (13)	0.0089 (15)	−0.0114 (11)	0.0078 (13)
C115	0.0510 (15)	0.0342 (12)	0.096 (2)	0.0194 (11)	−0.0111 (14)	0.0103 (13)
C108	0.0704 (19)	0.0515 (15)	0.0595 (16)	0.0213 (15)	−0.0239 (14)	−0.0203 (13)
C125	0.0434 (14)	0.0411 (14)	0.103 (3)	0.0135 (12)	−0.0251 (15)	0.0133 (14)

### Geometric parameters (Å, º)

**Table:**

O4—C9	1.203 (3)	C133—H133	0.9500
O101—C107	1.340 (3)	C133—C132	1.368 (5)
O101—C108	1.443 (3)	C117—C118	1.395 (3)
O103—C109	1.327 (3)	C117—C122	1.400 (3)
O103—C110	1.442 (3)	C16—H16	0.9500
O2—C7	1.204 (3)	C16—C15	1.389 (4)
O1—C7	1.331 (3)	C112—H112	0.9500
O1—C8	1.446 (3)	C112—C113	1.385 (3)
O3—C9	1.329 (3)	C130—H130	0.9500
O3—C10	1.451 (3)	C130—C131	1.396 (4)
O102—C107	1.196 (3)	C28—H28	0.9500
O104—C109	1.206 (3)	C28—C27	1.382 (4)
C9—C2	1.537 (3)	C124—H124	0.9500
C123—C105	1.493 (3)	C124—C125	1.388 (4)
C123—C124	1.395 (3)	C113—H113	0.9500
C123—C128	1.383 (3)	C113—C114	1.380 (4)
C29—C6	1.489 (3)	C119—H119	0.9500
C29—C34	1.390 (3)	C119—C118	1.389 (3)
C29—C30	1.396 (3)	C119—C120	1.384 (5)
C7—C1	1.524 (3)	C19—H19	0.9500
C4—C3	1.349 (3)	C19—C20	1.381 (4)
C4—C5	1.491 (3)	C131—H131	0.9500
C4—C17	1.496 (3)	C131—C132	1.381 (5)
C3—C2	1.529 (3)	C15—H15	0.9500
C3—C11	1.492 (3)	C15—C14	1.388 (4)
C23—C5	1.494 (3)	C118—H118	0.9500
C23—C24	1.390 (3)	C120—H120	0.9500
C23—C28	1.393 (3)	C120—C121	1.376 (4)
C109—C102	1.529 (3)	C31—H31	0.9500
C101—H101	0.99 (3)	C31—C32	1.375 (5)
C101—C107	1.514 (3)	C114—H114	0.9500
C101—C106	1.527 (3)	C114—C115	1.381 (4)
C101—C102	1.536 (3)	C116—H116	0.9500
C104—C105	1.493 (3)	C116—C115	1.390 (4)
C104—C103	1.356 (3)	C25—H25	0.9500
C104—C117	1.497 (3)	C25—C26	1.378 (5)
C105—C106	1.346 (3)	C27—H27	0.9500
C5—C6	1.348 (3)	C27—C26	1.374 (5)
C106—C129	1.490 (3)	C121—H121	0.9500
C102—H102	0.96 (3)	C121—C122	1.386 (4)
C102—C103	1.533 (3)	C8—H8A	0.9800
C2—H2	0.98 (3)	C8—H8B	0.9800
C2—C1	1.539 (3)	C8—H8C	0.9800
C6—C1	1.530 (3)	C122—H122	0.9500
C129—C134	1.391 (3)	C128—H128	0.9500
C129—C130	1.392 (3)	C128—C127	1.394 (4)
C11—C12	1.394 (3)	C13—H13	0.9500
C11—C16	1.393 (3)	C13—C14	1.376 (4)
C21—H21	0.9500	C20—H20	0.9500
C21—C22	1.391 (4)	C132—H132	0.9500
C21—C20	1.383 (4)	C26—H26	0.9500
C34—H34	0.9500	C14—H14	0.9500
C34—C33	1.388 (3)	C33—H33	0.9500
C18—H18	0.9500	C33—C32	1.376 (4)
C18—C17	1.397 (3)	C32—H32	0.9500
C18—C19	1.391 (3)	C110—H11A	0.9800
C103—C111	1.489 (3)	C110—H11B	0.9800
C111—C112	1.398 (3)	C110—H11C	0.9800
C111—C116	1.396 (3)	C10—H10A	0.9800
C30—H30	0.9500	C10—H10B	0.9800
C30—C31	1.393 (4)	C10—H10C	0.9800
C134—H134	0.9500	C126—H126	0.9500
C134—C133	1.392 (3)	C126—C127	1.368 (6)
C1—H1	0.98 (3)	C126—C125	1.373 (6)
C17—C22	1.392 (3)	C127—H127	0.9500
C24—H24	0.9500	C115—H115	0.9500
C24—C25	1.389 (4)	C108—H10D	0.9800
C22—H22	0.9500	C108—H10E	0.9800
C12—H12	0.9500	C108—H10F	0.9800
C12—C13	1.392 (3)	C125—H125	0.9500
			
C107—O101—C108	115.4 (2)	C15—C16—C11	120.6 (2)
C109—O103—C110	115.50 (19)	C15—C16—H16	119.7
C7—O1—C8	115.0 (2)	C111—C112—H112	119.5
C9—O3—C10	114.9 (2)	C113—C112—C111	120.9 (2)
O4—C9—O3	124.1 (2)	C113—C112—H112	119.5
O4—C9—C2	123.3 (2)	C129—C130—H130	119.9
O3—C9—C2	112.58 (18)	C129—C130—C131	120.2 (2)
C124—C123—C105	121.3 (2)	C131—C130—H130	119.9
C128—C123—C105	120.1 (2)	C23—C28—H28	119.5
C128—C123—C124	118.6 (2)	C27—C28—C23	120.9 (3)
C34—C29—C6	120.32 (19)	C27—C28—H28	119.5
C34—C29—C30	118.8 (2)	C123—C124—H124	120.0
C30—C29—C6	120.9 (2)	C125—C124—C123	120.0 (3)
O2—C7—O1	123.8 (2)	C125—C124—H124	120.0
O2—C7—C1	124.2 (2)	C112—C113—H113	119.7
O1—C7—C1	112.05 (18)	C114—C113—C112	120.6 (2)
C3—C4—C5	120.32 (19)	C114—C113—H113	119.7
C3—C4—C17	122.02 (19)	C118—C119—H119	119.9
C5—C4—C17	117.58 (18)	C120—C119—H119	119.9
C4—C3—C2	119.78 (18)	C120—C119—C118	120.1 (3)
C4—C3—C11	124.55 (19)	C18—C19—H19	120.0
C11—C3—C2	115.66 (18)	C20—C19—C18	120.0 (3)
C24—C23—C5	122.0 (2)	C20—C19—H19	120.0
C24—C23—C28	118.4 (2)	C130—C131—H131	120.0
C28—C23—C5	119.6 (2)	C132—C131—C130	120.0 (3)
O103—C109—C102	112.96 (17)	C132—C131—H131	120.0
O104—C109—O103	123.5 (2)	C16—C15—H15	119.8
O104—C109—C102	123.5 (2)	C14—C15—C16	120.4 (3)
C107—C101—H101	106.2 (15)	C14—C15—H15	119.8
C107—C101—C106	112.23 (17)	C117—C118—H118	119.6
C107—C101—C102	110.49 (17)	C119—C118—C117	120.8 (2)
C106—C101—H101	109.1 (15)	C119—C118—H118	119.6
C106—C101—C102	111.29 (17)	C119—C120—H120	120.2
C102—C101—H101	107.2 (16)	C121—C120—C119	119.7 (2)
C105—C104—C117	117.26 (18)	C121—C120—H120	120.2
C103—C104—C105	120.32 (18)	C30—C31—H31	120.0
C103—C104—C117	122.36 (19)	C32—C31—C30	120.0 (3)
C104—C105—C123	118.82 (18)	C32—C31—H31	120.0
C106—C105—C123	120.74 (18)	C113—C114—H114	120.4
C106—C105—C104	120.42 (18)	C113—C114—C115	119.2 (2)
O101—C107—C101	110.97 (18)	C115—C114—H114	120.4
O102—C107—O101	123.7 (2)	C111—C116—H116	119.8
O102—C107—C101	125.4 (2)	C115—C116—C111	120.5 (2)
C4—C5—C23	118.71 (18)	C115—C116—H116	119.8
C6—C5—C4	120.79 (19)	C24—C25—H25	119.7
C6—C5—C23	120.48 (18)	C26—C25—C24	120.6 (3)
C105—C106—C101	118.93 (18)	C26—C25—H25	119.7
C105—C106—C129	121.73 (18)	C28—C27—H27	119.9
C129—C106—C101	119.31 (17)	C26—C27—C28	120.3 (3)
C109—C102—C101	114.49 (17)	C26—C27—H27	119.9
C109—C102—H102	103.6 (16)	C120—C121—H121	119.6
C109—C102—C103	111.72 (17)	C120—C121—C122	120.7 (3)
C101—C102—H102	108.5 (16)	C122—C121—H121	119.6
C103—C102—C101	109.15 (17)	O1—C8—H8A	109.5
C103—C102—H102	109.1 (16)	O1—C8—H8B	109.5
C9—C2—H2	103.9 (16)	O1—C8—H8C	109.5
C9—C2—C1	113.86 (16)	H8A—C8—H8B	109.5
C3—C2—C9	111.29 (17)	H8A—C8—H8C	109.5
C3—C2—H2	108.7 (16)	H8B—C8—H8C	109.5
C3—C2—C1	109.97 (17)	C117—C122—H122	119.8
C1—C2—H2	108.8 (16)	C121—C122—C117	120.4 (2)
C29—C6—C1	118.19 (17)	C121—C122—H122	119.8
C5—C6—C29	122.85 (18)	C123—C128—H128	119.7
C5—C6—C1	118.93 (18)	C123—C128—C127	120.6 (3)
C134—C129—C106	119.5 (2)	C127—C128—H128	119.7
C134—C129—C130	118.7 (2)	C12—C13—H13	119.9
C130—C129—C106	121.7 (2)	C14—C13—C12	120.3 (2)
C12—C11—C3	120.1 (2)	C14—C13—H13	119.9
C16—C11—C3	121.59 (19)	C21—C20—H20	120.0
C16—C11—C12	118.3 (2)	C19—C20—C21	120.0 (2)
C22—C21—H21	120.0	C19—C20—H20	120.0
C20—C21—H21	120.0	C133—C132—C131	120.2 (2)
C20—C21—C22	120.0 (3)	C133—C132—H132	119.9
C29—C34—H34	119.9	C131—C132—H132	119.9
C33—C34—C29	120.2 (2)	C25—C26—H26	120.2
C33—C34—H34	119.9	C27—C26—C25	119.6 (2)
C17—C18—H18	119.6	C27—C26—H26	120.2
C19—C18—H18	119.6	C15—C14—H14	120.3
C19—C18—C17	120.8 (2)	C13—C14—C15	119.5 (2)
C104—C103—C102	119.08 (18)	C13—C14—H14	120.3
C104—C103—C111	124.94 (19)	C34—C33—H33	119.7
C111—C103—C102	115.87 (18)	C32—C33—C34	120.6 (2)
C112—C111—C103	120.8 (2)	C32—C33—H33	119.7
C116—C111—C103	120.99 (19)	C31—C32—C33	120.0 (2)
C116—C111—C112	118.0 (2)	C31—C32—H32	120.0
C29—C30—H30	119.8	C33—C32—H32	120.0
C31—C30—C29	120.4 (2)	O103—C110—H11A	109.5
C31—C30—H30	119.8	O103—C110—H11B	109.5
C129—C134—H134	119.8	O103—C110—H11C	109.5
C129—C134—C133	120.5 (2)	H11A—C110—H11B	109.5
C133—C134—H134	119.8	H11A—C110—H11C	109.5
C7—C1—C2	110.82 (17)	H11B—C110—H11C	109.5
C7—C1—C6	110.29 (16)	O3—C10—H10A	109.5
C7—C1—H1	107.3 (16)	O3—C10—H10B	109.5
C2—C1—H1	107.5 (16)	O3—C10—H10C	109.5
C6—C1—C2	111.88 (17)	H10A—C10—H10B	109.5
C6—C1—H1	108.9 (16)	H10A—C10—H10C	109.5
C18—C17—C4	121.7 (2)	H10B—C10—H10C	109.5
C22—C17—C4	119.9 (2)	C127—C126—H126	120.2
C22—C17—C18	118.3 (2)	C127—C126—C125	119.6 (3)
C23—C24—H24	119.9	C125—C126—H126	120.2
C25—C24—C23	120.2 (3)	C128—C127—H127	119.8
C25—C24—H24	119.9	C126—C127—C128	120.4 (3)
C21—C22—C17	120.8 (3)	C126—C127—H127	119.8
C21—C22—H22	119.6	C114—C115—C116	120.8 (2)
C17—C22—H22	119.6	C114—C115—H115	119.6
C11—C12—H12	119.6	C116—C115—H115	119.6
C13—C12—C11	120.9 (2)	O101—C108—H10D	109.5
C13—C12—H12	119.6	O101—C108—H10E	109.5
C134—C133—H133	119.9	O101—C108—H10F	109.5
C132—C133—C134	120.3 (3)	H10D—C108—H10E	109.5
C132—C133—H133	119.9	H10D—C108—H10F	109.5
C118—C117—C104	121.9 (2)	H10E—C108—H10F	109.5
C118—C117—C122	118.2 (2)	C124—C125—H125	119.6
C122—C117—C104	119.8 (2)	C126—C125—C124	120.8 (3)
C11—C16—H16	119.7	C126—C125—H125	119.6
			
O4—C9—C2—C3	112.9 (2)	C102—C103—C111—C116	−42.2 (3)
O4—C9—C2—C1	−122.1 (2)	C2—C3—C11—C12	−129.8 (2)
O103—C109—C102—C101	−52.7 (2)	C2—C3—C11—C16	47.6 (3)
O103—C109—C102—C103	72.0 (2)	C6—C29—C34—C33	−179.7 (2)
O2—C7—C1—C2	−44.6 (3)	C6—C29—C30—C31	179.8 (2)
O2—C7—C1—C6	79.8 (3)	C129—C134—C133—C132	0.0 (4)
O1—C7—C1—C2	136.50 (18)	C129—C130—C131—C132	−1.1 (4)
O1—C7—C1—C6	−99.1 (2)	C11—C3—C2—C9	−89.4 (2)
O3—C9—C2—C3	−64.5 (2)	C11—C3—C2—C1	143.46 (19)
O3—C9—C2—C1	60.5 (2)	C11—C12—C13—C14	−0.3 (4)
O104—C109—C102—C101	129.4 (2)	C11—C16—C15—C14	0.5 (4)
O104—C109—C102—C103	−105.9 (2)	C34—C29—C6—C5	59.7 (3)
C9—C2—C1—C7	46.1 (2)	C34—C29—C6—C1	−118.4 (2)
C9—C2—C1—C6	−77.4 (2)	C34—C29—C30—C31	−1.3 (3)
C123—C105—C106—C101	−179.44 (18)	C34—C33—C32—C31	−1.0 (4)
C123—C105—C106—C129	−1.4 (3)	C18—C17—C22—C21	0.2 (3)
C123—C124—C125—C126	0.6 (4)	C18—C19—C20—C21	0.1 (4)
C123—C128—C127—C126	−0.5 (4)	C103—C104—C105—C123	163.0 (2)
C29—C6—C1—C7	21.4 (3)	C103—C104—C105—C106	−15.3 (3)
C29—C6—C1—C2	145.28 (18)	C103—C104—C117—C118	132.3 (2)
C29—C34—C33—C32	−0.2 (4)	C103—C104—C117—C122	−51.8 (3)
C29—C30—C31—C32	0.2 (4)	C103—C111—C112—C113	−174.8 (2)
C4—C3—C2—C9	91.4 (2)	C103—C111—C116—C115	175.3 (3)
C4—C3—C2—C1	−35.7 (3)	C111—C112—C113—C114	0.3 (4)
C4—C3—C11—C12	49.4 (3)	C111—C116—C115—C114	−1.2 (5)
C4—C3—C11—C16	−133.2 (3)	C30—C29—C6—C5	−121.4 (2)
C4—C5—C6—C29	−177.45 (19)	C30—C29—C6—C1	60.5 (3)
C4—C5—C6—C1	0.7 (3)	C30—C29—C34—C33	1.4 (3)
C4—C17—C22—C21	176.8 (2)	C30—C31—C32—C33	1.0 (4)
C3—C4—C5—C23	−163.1 (2)	C134—C129—C130—C131	1.7 (3)
C3—C4—C5—C6	15.2 (3)	C134—C133—C132—C131	0.6 (4)
C3—C4—C17—C18	−132.3 (2)	C17—C4—C3—C2	−172.26 (19)
C3—C4—C17—C22	51.2 (3)	C17—C4—C3—C11	8.6 (3)
C3—C2—C1—C7	171.79 (17)	C17—C4—C5—C23	13.6 (3)
C3—C2—C1—C6	48.2 (2)	C17—C4—C5—C6	−168.1 (2)
C3—C11—C12—C13	178.3 (2)	C17—C18—C19—C20	0.4 (3)
C3—C11—C16—C15	−178.3 (2)	C24—C23—C5—C4	−122.2 (2)
C23—C5—C6—C29	0.8 (3)	C24—C23—C5—C6	59.5 (3)
C23—C5—C6—C1	178.93 (18)	C24—C23—C28—C27	−0.3 (3)
C23—C24—C25—C26	−0.9 (4)	C24—C25—C26—C27	0.5 (4)
C23—C28—C27—C26	−0.1 (4)	C22—C21—C20—C19	−0.5 (4)
C109—C102—C103—C104	−89.5 (2)	C12—C11—C16—C15	−0.9 (4)
C109—C102—C103—C111	94.1 (2)	C12—C13—C14—C15	−0.1 (4)
C101—C106—C129—C134	115.8 (2)	C117—C104—C105—C123	−14.3 (3)
C101—C106—C129—C130	−64.9 (3)	C117—C104—C105—C106	167.5 (2)
C101—C102—C103—C104	38.2 (3)	C117—C104—C103—C102	171.84 (19)
C101—C102—C103—C111	−138.24 (19)	C117—C104—C103—C111	−12.1 (3)
C104—C105—C106—C101	−1.3 (3)	C16—C11—C12—C13	0.8 (4)
C104—C105—C106—C129	176.77 (18)	C16—C15—C14—C13	0.1 (4)
C104—C103—C111—C112	−43.7 (3)	C112—C111—C116—C115	0.5 (4)
C104—C103—C111—C116	141.7 (2)	C112—C113—C114—C115	−1.0 (4)
C104—C117—C118—C119	176.6 (2)	C130—C129—C134—C133	−1.2 (3)
C104—C117—C122—C121	−175.8 (2)	C130—C131—C132—C133	−0.1 (4)
C105—C123—C124—C125	177.7 (2)	C28—C23—C5—C4	58.8 (3)
C105—C123—C128—C127	−177.8 (2)	C28—C23—C5—C6	−119.5 (2)
C105—C104—C103—C102	−5.2 (3)	C28—C23—C24—C25	0.8 (3)
C105—C104—C103—C111	170.81 (19)	C28—C27—C26—C25	0.0 (4)
C105—C104—C117—C118	−50.5 (3)	C124—C123—C105—C104	122.2 (2)
C105—C104—C117—C122	125.3 (2)	C124—C123—C105—C106	−59.6 (3)
C105—C106—C129—C134	−62.2 (3)	C124—C123—C128—C127	0.7 (3)
C105—C106—C129—C130	117.1 (2)	C113—C114—C115—C116	1.4 (5)
C107—C101—C106—C105	159.64 (19)	C119—C120—C121—C122	0.2 (4)
C107—C101—C106—C129	−18.4 (3)	C19—C18—C17—C4	−177.1 (2)
C107—C101—C102—C109	−50.6 (2)	C19—C18—C17—C22	−0.6 (3)
C107—C101—C102—C103	−176.70 (17)	C118—C117—C122—C121	0.2 (3)
C5—C4—C3—C2	4.3 (3)	C118—C119—C120—C121	0.7 (4)
C5—C4—C3—C11	−174.8 (2)	C120—C119—C118—C117	−1.2 (4)
C5—C4—C17—C18	51.0 (3)	C120—C121—C122—C117	−0.6 (4)
C5—C4—C17—C22	−125.4 (2)	C116—C111—C112—C113	−0.1 (3)
C5—C23—C24—C25	−178.2 (2)	C8—O1—C7—O2	−7.3 (3)
C5—C23—C28—C27	178.7 (2)	C8—O1—C7—C1	171.6 (2)
C5—C6—C1—C7	−156.78 (19)	C122—C117—C118—C119	0.7 (3)
C5—C6—C1—C2	−32.9 (3)	C128—C123—C105—C104	−59.4 (3)
C106—C101—C107—O101	91.5 (2)	C128—C123—C105—C106	118.9 (2)
C106—C101—C107—O102	−87.5 (3)	C128—C123—C124—C125	−0.7 (3)
C106—C101—C102—C109	74.7 (2)	C20—C21—C22—C17	0.4 (4)
C106—C101—C102—C103	−51.3 (2)	C110—O103—C109—O104	−7.8 (3)
C106—C129—C134—C133	178.2 (2)	C110—O103—C109—C102	174.3 (2)
C106—C129—C130—C131	−177.7 (2)	C10—O3—C9—O4	9.6 (3)
C102—C101—C107—O101	−143.65 (18)	C10—O3—C9—C2	−173.1 (2)
C102—C101—C107—O102	37.3 (3)	C127—C126—C125—C124	−0.4 (4)
C102—C101—C106—C105	35.2 (3)	C108—O101—C107—O102	5.3 (3)
C102—C101—C106—C129	−142.85 (18)	C108—O101—C107—C101	−173.7 (2)
C102—C103—C111—C112	132.4 (2)	C125—C126—C127—C128	0.3 (4)

### Hydrogen-bond geometry (Å, º)

**Table:**

*D*—H···*A*	*D*—H	H···*A*	*D*···*A*	*D*—H···*A*
C101—H101···O2	0.99 (3)	2.39 (3)	3.384 (3)	176 (2)
C102—H102···O4	0.96 (3)	2.48 (3)	3.242 (3)	136 (2)
C16—H16···O4	0.95	2.59	3.407 (3)	145
C116—H116···O104	0.95	2.54	3.388 (3)	148
